# Supplementary figures and images for: Pain, fatigue, and associated gene expressions over chemotherapy in patients with colorectal cancer
Source: PLoS One. 2025 Jun 27;20(6):e0325849. doi: 10.1371/journal.pone.0325849 (PMC12204541; doi:10.1371/journal.pone.0325849)

**RNA Sequencing Analysis Workflow**

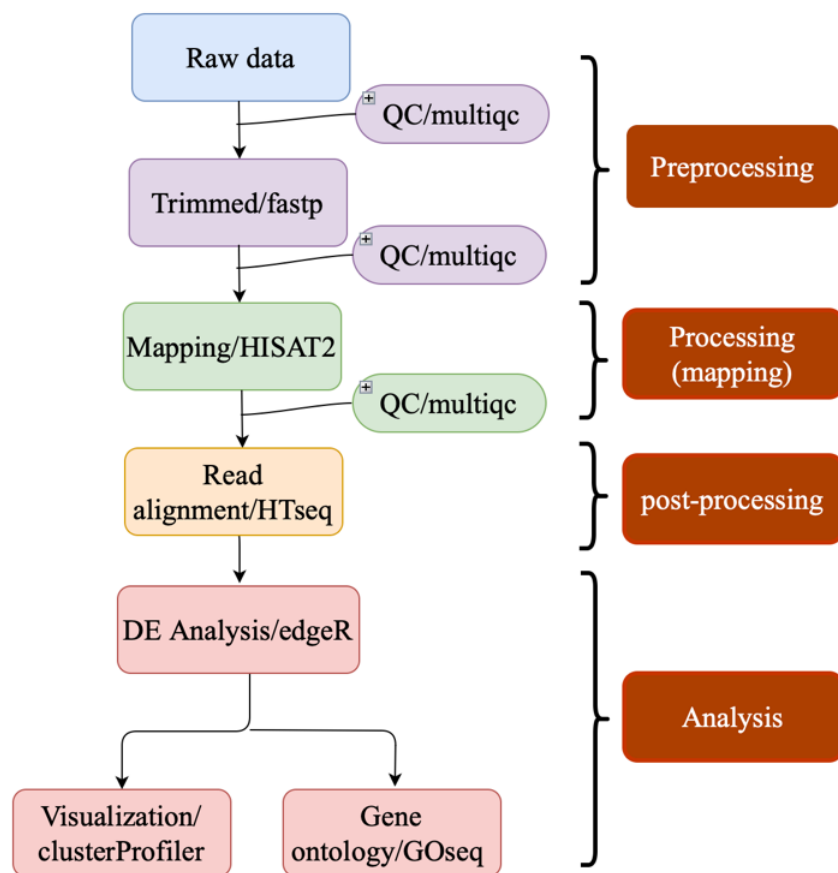

S1 Fig 1: The RNA seq Analysis Workflow.

Supplement: S1 Fig 1 — The RNA sequencing differential expression (DE) analysis comprised four main stages: preprocessing, mapping, post-processing, and data analysis. During preprocessing, the data underwent quality assessment pre- and post-trimming using fastqc and multiqc, as well as trimming using fastp. In the mapping stage, we selected the human genome hg19 as the mapping index and aligned the reads to the reference genome with HISTA. For post-processing, the program HTseq-count was used to count the RNA fragments (i.e., read pairs) mapped to each annotated gene in the genome. Once the counts were generated, we applied R package edgeR to perform gene DE analysis. Enrichment analysis was performed using the GOseq packages. Mean-difference (MD) plots and enrichment dot plots illustrated the results for visualization by clusterProfiler package. (PDF) [file pone.0325849.s001.pdf]
