# Supplementary material for: Pain, fatigue, and associated gene expressions over chemotherapy in patients with colorectal cancer
Source: PLoS One. 2025 Jun 27;20(6):e0325849. doi: 10.1371/journal.pone.0325849 (PMC12204541; doi:10.1371/journal.pone.0325849)
Supplement: S1 Table 1 — (PDF) [file pone.0325849.s002.pdf]

S1 Table 1. Differentially expressed gene list (logFC > 1.5 & FDR < 0.05)

**Visit 2 versus Visit 1**				**Visit 3 versus Visit 2**			
Genes	logFC	PValue	FDR	Genes	logFC	PValue	FDR
JCHAIN	-1.939	9.17E-16	6.73E-12	SLC4A3	1.682	1.76E-12	7.61E-09
TOP2A	-1.922	3.80E-09	8.24E-06	JCHAIN	2.183	7.58E-11	1.64E-07
RRM2	-2.003	1.01E-07	9.40E-05	IGHG1	2.366	1.47E-10	2.73E-07
ACHE	-1.862	1.11E-07	9.65E-05	SYN2	-1.782	9.01E-10	1.06E-06
ALOX15	-2.162	1.39E-06	0.00058138	ALAS2	1.759	8.13E-09	6.20E-06
IGHG1	-1.991	7.86E-06	0.00179247	MKI67	2.124	1.03E-08	6.73E-06
IGHA1	-1.553	1.42E-05	0.00262241	BIRC5	2.300	1.44E-08	8.86E-06
LYPD2	-1.691	2.62E-05	0.0038324	SLPI	-1.692	1.67E-08	9.41E-06
OLAH	2.031	2.77E-05	0.00395483	SLC6A9	2.256	2.84E-08	1.31E-05
ADAMTS2	2.582	5.30E-05	0.00577983	TOP2A	1.889	4.07E-08	1.56E-05
AP3B2	1.533	8.23E-05	0.00747965	CCNB2	1.612	4.10E-08	1.56E-05
CDK1	-1.646	9.08E-05	0.00780542	RRM2	2.058	7.00E-08	2.26E-05
SIGLEC8	-1.607	0.00013173	0.00955428	ACHE	1.836	3.50E-07	6.46E-05
MKI67	-1.727	0.00024193	0.01422557	IGHA1	2.001	9.44E-07	0.0001275
CACNG6	-1.595	0.00034484	0.01730168	DNASE1L3	1.763	2.02E-06	0.00022392
IL5RA	-1.504	0.00050478	0.02111301	IGLC3	1.537	2.18E-06	0.00023601
LILRA6.1	1.638	0.00060235	0.02329112	PLD4	1.619	2.34E-06	0.00024622
ZDHHC19	1.816	0.00070815	0.02570489	ALOX15	2.286	3.93E-06	0.00035611
CEACAM8	-1.503	0.0008929	0.02952478	FFAR3	-1.577	4.27E-06	0.00037717
LOC105377267	-1.504	0.00099944	0.03200567	TNFRSF17	1.671	2.43E-05	0.00130084
SLC6A9	-1.524	0.00133083	0.03799486	IGHG2	2.675	4.99E-05	0.00208216
IGHG2	-2.083	0.00188973	0.04612144	SIGLEC8	1.665	9.57E-05	0.00321729
				RAP1GAP	1.508	0.00024861	0.00602869
				LYPD2	1.609	0.00059796	0.01132204
				CACNG6	1.601	0.00067192	0.01218848
				DAAM2	-1.534	0.00100182	0.01589995
				PRSS33	1.690	0.00118444	0.01755671
				IGLV1-44	1.676	0.00502307	0.04607443
