# Supplementary material for: Pain, fatigue, and associated gene expressions over chemotherapy in patients with colorectal cancer
Source: PLoS One. 2025 Jun 27;20(6):e0325849. doi: 10.1371/journal.pone.0325849 (PMC12204541; doi:10.1371/journal.pone.0325849)
Supplement: S1 Fig 2 — Individuals who reported higher pain scores at visit 2 compared to visit 1 were classified as belonging to Pain Trajectory 1, while those who reported lower pain scores were classified as Pain Trajectory 2. In a similar way, individuals who experienced increased fatigue levels at visit 2 were categorized as being in Fatigue Trajectory 1, whereas those with decreased fatigue levels were placed in Fatigue Trajectory 2. (PDF) [file pone.0325849.s003.pdf]

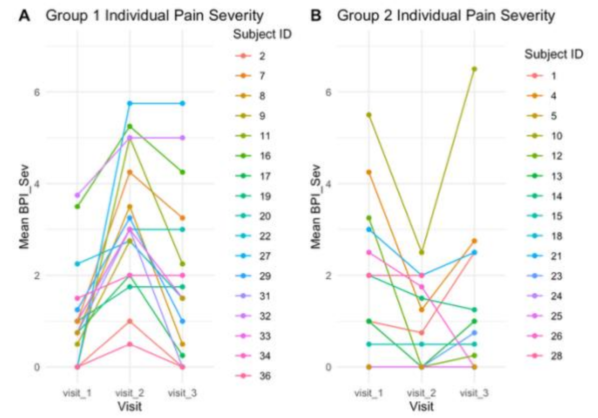

S1 Fig 2a. Individual Pain Severity Over

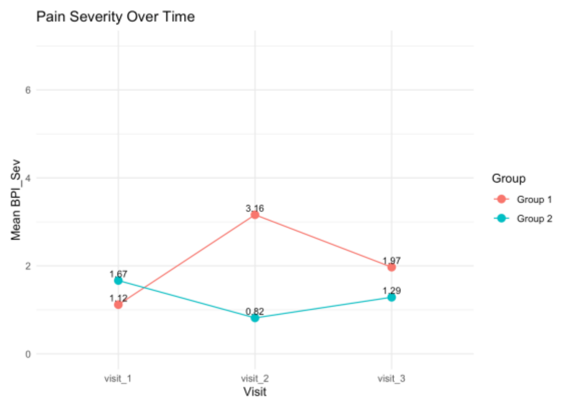

S1 Fig 2b. Group Mean of Pain Severity Over Time

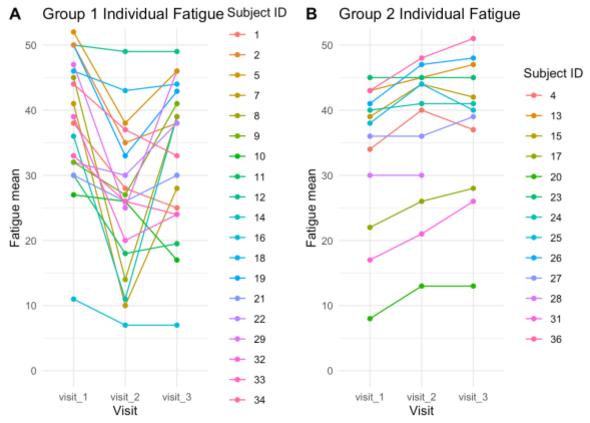

S1 Fig 2c. Individual Fatigue Over Time

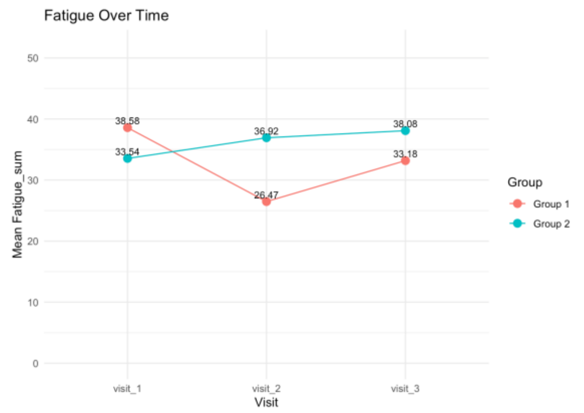

S1 Fig 2d. Group Mean of Fatigue Over Time

S1 Fig 2. Individual symptom trajectories and mean scores among distinct pain/fatigue trajectories.
