# Supplementary material for: Pain, fatigue, and associated gene expressions over chemotherapy in patients with colorectal cancer
Source: PLoS One. 2025 Jun 27;20(6):e0325849. doi: 10.1371/journal.pone.0325849 (PMC12204541; doi:10.1371/journal.pone.0325849)
Supplement: Attachment — Submitted filename: comments 10.docx [file pone.0325849.s004.docx]

**Praising the Article**

The manuscript titled "Pain, Fatigue, and Associated Gene Expressions Over Chemotherapy in Patients with Colorectal Cancer" presents a compelling and timely investigation into the dynamic changes in pain, fatigue, and associated gene expressions in colorectal cancer patients undergoing chemotherapy. The longitudinal study design is commendable, and the integration of self-reported symptom assessments with RNA sequencing provides valuable insights into the biological underpinnings of chemotherapy-induced symptom burden. The study’s focus on inflammatory pathways and their relationship with pain and fatigue enhances its significance in oncology and symptom management research.

The manuscript is well-structured, and the authors effectively contextualize their findings within the existing literature. The statistical approaches used for differential gene expression analysis and linear mixed models are appropriate for the study design. The discussion section highlights important implications for future research and potential clinical applications. However, there are major concerns about the current version of this manuscript as described below.

### ****Major Concerns:****

- The study includes a relatively small sample size (n=34), which limits the generalizability of the findings. Given the complexity of pain and fatigue mechanisms, a larger cohort is needed to confirm the associations between symptom trajectories and gene expression changes.
- The demographic homogeneity of the sample (97% white and non-Hispanic) further restricts the applicability of the results to more diverse populations.
- While the authors perform appropriate statistical tests, the interpretation of certain results is overly strong given the small sample size. For example, the associations between LILRA6 expression and pain/fatigue (p = 0.010) and between PRSS33 upregulation and pain interference (p = 0.038) should be reported with caution, acknowledging the potential for Type I error.
- The post-hoc analysis for symptom changes over time lacks a clear adjustment for multiple comparisons. A more conservative approach, such as Bonferroni correction, may be needed.
- The discussion effectively links the findings to immune-inflammatory pathways, but it lacks a mechanistic explanation of how chemotherapy-induced inflammation translates into altered gene expression patterns. Including references to experimental studies on the molecular pathways of pain and fatigue could strengthen this section.
- The role of the identified genes (e.g., LILRA6, CACNG6, and PRSS33) in pain and fatigue needs further validation. The authors should discuss whether these genes have been implicated in previous research on cancer-related symptoms.
- Figures 2 and 3 illustrating gene expression changes and pathway enrichment are informative but somewhat difficult to interpret. Providing clearer legends and emphasizing the key findings in the text would improve comprehension.
- The trajectory classification for pain and fatigue appears somewhat arbitrary. The authors should clarify the criteria used to distinguish between different trajectory groups and consider alternative clustering methods for greater robustness.

### ****Minor Concerns:****

- The manuscript sometimes alternates between ‘chemotherapy’ and ‘CTX.’ It would be helpful to standardize the terminology throughout.
- The abbreviation ‘LMM’ (Linear Mixed Models) is introduced without a clear explanation. A brief clarification in the methods section would be beneficial.
- The manuscript states that ‘some restrictions apply’ to data availability. The authors should provide more details on how interested researchers can access the dataset and under what conditions.
